# Development and characterization of a chicory extract fermented by *Akkermansia muciniphila*: An *in vitro* study on its potential to modulate obesity-related inflammation

**DOI:** 10.1016/j.crfs.2025.100974

**Published:** 2025-01-16

**Authors:** A. Chervet, R. Nehme, C. Defois-Fraysse, C. Decombat, C. Blavignac, C. Auxenfans, B. Evrard, S. Michel, E. Filaire, J.-Y. Berthon, A. Dreux-Zigha, L. Delort, F. Caldefie-Chézet

**Affiliations:** a Université Clermont-Auvergne, INRAE, UNH, Unité de Nutrition Humaine, CRNH-Auvergne, 63000, Clermont-Ferrand, France; b Greencell, Biopôle Clermont-Limagne, 63360, Saint-Beauzire, France; c Université Clermont-Auvergne, Centre d’Imagerie Cellulaire Santé (CCIS), Clermont-Ferrand, France; d Banque de Tissus et de Cellules, Hôpital Edouard-Herriot, 69000, Lyon, France; e Greentech, Biopôle Clermont-Limagne, 63360, Saint-Beauzire, France

**Keywords:** *Akkermansia muciniphila*, Fermentation, Prebiotics, Postbiotics, Obesity, Inflammation

## Abstract

Obesity, the fifth leading cause of death globally and linked to chronic low-grade inflammation and development of numerous severe pathologies, is a major public health problem. Fermented foods, probiotics, and postbiotics emerge as promising avenues for combating obesity and inflammation. The aim of our study was to develop and characterize phyto-postbiotics corresponding to prebiotic compounds fermented by gut bacteria, which could act on obesity and related-inflammation. Chicory extract fermented by *Akkermansia muciniphila* (C-Akm) was selected as the most antioxidant of 20 fermented extracts. The identification of metabolites derived from C-Akm extract has enabled us to detect mostly amino acids, acids, and some polyphenols (daidzein and genistein). The anti-inflammatory and anti-obesity activities of C-Akm extract were studied by testing the extract (50 μg/mL) on the polarization of THP-1 into macrophages, the secretion of pro-inflammatory cytokines in LPS-stimulated PBMCs, and the secretion of leptin and adiponectin in adipospheroids derived from human adipose stem cells. Finally, the extract was examined in 3D co-culture model mimicking inflamed obese adipose tissue. We found that C-Akm extract decreased ROS generation, *TNF-α* and *Il-6* gene expression in polarized macrophages, INFγ and IL-17A secretion in LPS-stimulated PBMCs stimulated with LPS. It also decreased *leptin* expression while increasing *adiponectin* and *HSL* expression levels in both adipocytes and co-cultures. In addition, C-Akm extract stimulated adiponectin secretion in the co-culture model. Finally, our *in vitro* investigations demonstrated the potential benefits of C-Akm extract in the prevention and treatment of obesity-related inflammation.

## Highlights

• A chicory extract fermented by *Akkermansia muciniphila* (C-Akm) was developed.
• C-Akm presented potent antioxidant and anti-inflammatory effects.
• C-Akm could enhanced adiponectin level in human obese mature adipospheroids.
• C-Akm could be a promising therapeutic agent for obesity-related inflammation.

## Graphical abstract

**Figure:**
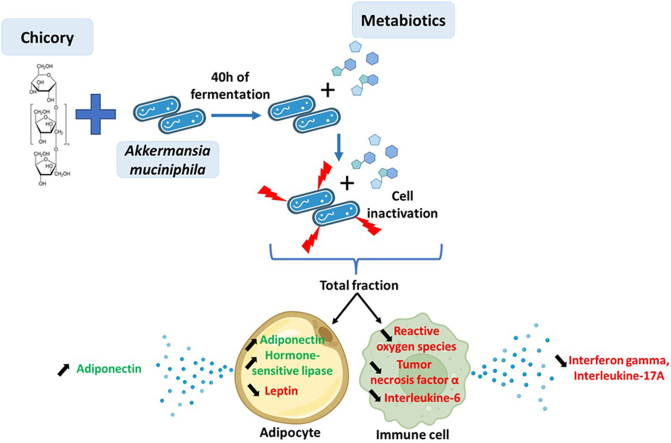

## Abbreviations:

AdipoR1: Adiponectin receptor 1
AdipoR2: Adiponectin receptor 2
APMK: Adenosine monophosphate activated protein kinase
AREs: Antioxidant response elements
ARG1: Arginase 1
BCCA: Branched chain amino acid
BMI: Body mass index
CD163: Cluster of differenciation 163
CD206: Cluster of differenciation 206
CFU: Colony forming units
COX-2: Cyclooxygenase-2 inhibitor
CXCL10: Human C-X-X motif chemokine ligand 10
Dhr: Dihydrorhodamine
DMEM: Dulbecco's Modified Eagle Medium
EDTA: Ethylenediaminetetraacetic acid
FBS: Fetal bovine serum
GAPDH: Glyceraldhehyde 3-phosphate dehydrogenase
Gln: Glutamine
GLP-1: Glucagon-like peptide-1
GSH-Px: Glutathione peroxidase
HPLC: High performance liquid chromatography
HSL: Hormone-sensitive lipase
IBMX: 3-isobutyl-1-methylxanthine
IFNy: Interferon gamma
IL-10: Interleukine-10
IL-17A: Interleukine-17A
IL1-β: Interleukine 1-beta
IL-2: Interleukine-2
IL-6: Interleukine-6
IL-6R: Interleukine-6 receptor
IL-8: Interleukine-8
JAK: Janus kinase
LPS: Lipopolysaccharides
MAPK: Mitogen activated protein kinase
MCP-1: Monocyte chemoattractant protein-1
MRS: Man, Rogosa, Sharpe
NF-κB: Nuclear factor kappa B
Nrf2: Nuclear factor erythroid 2-related factor 2
PBMC: Peripheral blood mononuclear cells
PC1: Preculture 1
PC2: Preculture 2
PMA: Phorbol myristate acetate
PPARα: Peroxisome proliferator activated receptor alpha
PPARγ: Peroxisome proliferator activated receptor gamma
ROS: Reactive oxygen species
RPMI: Roswell park memorial institute
RQ: Relative quantification
SCFA: Short chain fatty acids
SEM: Standard error of measurement
SOD: Superoxyde dismutase
STAT-1: Signal transducer and activator of transcription 1
T3: Triiodothyronine
TGF-β: Transforming growth factor beta
Th1: T helper cell type 1
Th2: T helper cell type 2
THP-1: Human leukemia monocytic cell line
TLR2: Toll-like receptor 2
TLR4: Toll-like receptor 4
TNFR: Tumor necrosis factor receptor
TNF-α: Tumor necrosis factor alpha
TS: Tryptone soja

## 1 Introduction

Obesity, which has become a global public health issue, is defined by an excessive accumulation of body fat that may impair health. It is a chronic disease and a major risk factor for many pathologies (Pi-Sunyer, 2009). The epidemiology of obesity demonstrates that its prevalence has gradually increased over the last few decades (Lingvay et al., 2024). According to WHO estimates, more than 2.5 billion persons are estimated to be overweight in 2022, where 890 million of these were obese (Obesity and overweight n). In obese people, adipose cells are characterized by a hypertrophy and a hyperplasia which endure higher cellular stress associated with modifications in adipose tissue vascularization (Longo et al., 2019). The local immune cell population will shift as the amount of fat tissue increases. Indeed, in a non-pathological situation, macrophages resident in adipose tissue represent ∼5–10% of total cells and express genes associated with a M2-type phenotype, such as CD163, CD206, Arginase 1 (ARG1) and transforming growth factor beta (TGF-β). On the contrary, in obesity, they account for up to 50% of all cells and are associated with a pro-inflammatory M1 phenotype (Weisberg et al., 2003; Castoldi et al., 2015). These pro-inflammatory cells are recruited in response to chemokines such as monocyte chemotactic protein-1 (MCP-1) and pro-inflammatory cytokines such as tumor necrosis factor alpha (TNF-α) and interleukin (IL)-6 produced by hypertrophic adipocytes and resident immune cells (Bai and Sun, 2015). In addition to immune cells, adipocytes will demonstrate mitochondrial breakdown resulting in oxidative stress and increased reactive oxygen species (ROS) generation, which are responsible for the expansion of inflammation (de Mello et al., 2018). In general, a deregulation of ROS production is responsible for the development of several serious diseases such as aging, inflammation, cancer, diabetes, and cardiovascular disease (Checa and Aran, 2020). These pro-inflammatory cytokines and ROS act as key mediators in the inflammatory cascade, activating various cellular signaling pathways and thus contributing to the establishment of a chronic inflammatory state and adipose tissue dysfunction associated with obesity (Kawai et al., 2021; Ellulu et al., 2017). In accordance with the increasing level of pro-inflammatory cytokines, adipokine production will be dysregulated, resulting in a significant increase in circulating leptin levels and a reduction in adiponectin (Obradovic et al., 2021; Achari and Jain, 2017). Leptin is known to exert its action by binding to its specific receptors (Ob-R) present in various parts of the body, including immune cells. In the context of inflammation, leptin plays an important role in stimulating immune cells such as T lymphocytes and macrophages, mainly *via* the JAK (Janus kinases)/STAT (signal transducers and activators of transcription) pathway, thereby promoting the production of pro-inflammatory cytokines such as IL-6 and TNF-α. Furthermore, it can alter the balance of Th1 and Th2 responses, altering the scale of the inflammatory reaction (Obradovic et al., 2021; Pérez-Pérez et al., 2020; Caldefie-Chezet et al., 2013). On the other hand, adiponectin binds to its specific receptors known as AdipoR1 and AdipoR2. Adiponectin primarily activates the AMP-activated protein kinase (AMPK) and peroxisome proliferator-activated receptor alpha (PPAR-α) signaling pathways. Activating these pathways improves insulin sensitivity, increases fatty acid oxidation, and decreases production of pro-inflammatory cytokines including TNF-α and IL-6 (Choi et al., 2020; Jarde et al., 2011). Adiponectin additionally enhances the production of anti-inflammatory cytokines like IL-10 (Wolf et al., 2004).

Many gut bacteria have been found to provide health benefits over time, particularly in relation to metabolic illnesses (Miao et al., 2023; Joung et al., 2021; O'Morain et al., 2021). Indeed, one of the most well-known Gram-negative anaerobic bacteria, *Akkermansia muciniphila*, has been linked to improved insulin sensitivity, which is beneficial for the management of type 2 diabetes (Zhao et al., 2017). This bacteria also appears to regulate lipid metabolism, which may assist to lower cholesterol levels (Si et al., 2022). Some studies show that this bacteria could improve body composition and help prevent obesity (Xu et al., 2020). Furthermore, prebiotics are non-digestible compounds that promote the development and activity of beneficial bacteria in the gut microbiome. The fermentation of polysaccharides by healthy gut bacteria has several health advantages, notably in helping to fight against obesity and inflammation (Zhang et al., 2018). The fermentation process produces secondary metabolites such as short-chain fatty acids (SCFAs), vitamins, proteins, amino acids, contributing to beneficial biological outcomes (Zhang et al., 2018). Furthermore, emerging research suggests that plant-based products could provide a more effective approach to treating and preventing a wide range of diseases (Alsahli M et al., 2021; Chervet et al., 2023).

The aim of this study was to develop, characterize, and compare extracts called phyto-postbiotics obtained after the fermentation between prebiotics (chicory or horse chestnut) and beneficial intestinal bacteria (10 strains) in order to study their biological effects on the modulation of the metabolism found in an obese situation, i.e. in a microenvironment reflecting an inflamed obese adipose tissue. The intestinal bacteria were heat-inactivated following the fermentation leading to the obtention of postbiotic extracts encompassing the bacterial metabolites from the fermentation process and the killed bacterial cells. In this study, we first investigated the antioxidant effect of 20 combinations (prebiotics/beneficial intestinal bacteria) on the ROS production by PMA-stimulated human leukocytes. The most active combination being *Akkermansia muciniphila*/Chicory (C-Akm), we opted to use this extract in the further course of the investigation. We hypothesized that the antioxidant capabilities of C-Akm extract would interact with the inflammatory response of immune cells and adipocytes. Finally, our study could reveal the value of such an extract in the prevention and management of inflammation-associated obesity.

## 2 Materials and methods

### 2.1 Generation of phyto-postbiotics

A combination of prebiotics (chicory or horse chestnut) and bacterial strains of interest (Table 1) were tested leading to the production of 20 freeze-dried phyto-postbiotics corresponding to the mixture of cultured supernatant and inactivated cells. Anaerobic strains were handled in an anaerobic chamber during all growth stages (Bactron 300, Kentron Microbiology, Doetinchem, Netherlands).

**Table 1:** Origin of the bacterial strains used.

Strain	Reference	Supplier
*Lactobacillus reuteri*	DSMZ 20016	Leibniz Institute DSMZ-German Collection of Microorganisms and Cell Cultures GmbH
*Lactobacillus gasseri*	DSMZ 20604	Leibniz Institute DSMZ-German Collection of Microorganisms and Cell Cultures GmbH
*Lactobacillus crispatus*	LMG 12005	Belgian Co-ordinated Collections of Micro-organisms (BCCM)
*Bacillus coagulans*	LMG 6326	Belgian Co-ordinated Collections of Micro-organisms (BCCM)
*Bifidobacterium longum*	CIP 64.62T	Institut Pasteur
*Bifidobacterium bifidum*	Internal isolation (human feces)	Greencell
*Bifidobacterium breve*	CIP 64.69T	Institut Pasteur
*Akkermansia muciniphila*	DSMZ - DSM 22959	Leibniz Institute DSMZ-German Collection of Microorganisms and Cell Cultures GmbH
*Faecalibacterium prausnitzii*	DSMZ DSM 17677	Leibniz Institute DSMZ-German Collection of Microorganisms and Cell Cultures GmbH
*Roseburia intestinalis*	DSMZ - DSM 14610	Leibniz Institute DSMZ-German Collection of Microorganisms and Cell Cultures GmbH

**Fig. 1:**
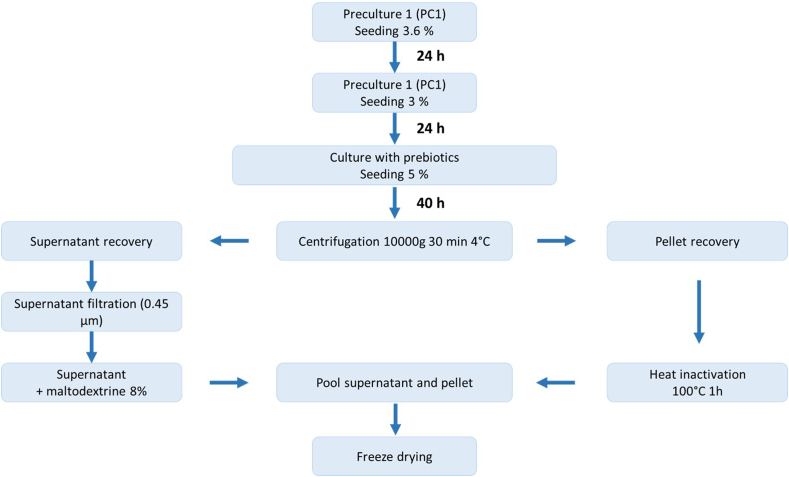
Generation of phyto-postbiotics.

Briefly, bacteria were seeded from glycerol stocks at 3.6 % (preculture 1 = PC1) either in TS (tryptone soja) or MRS (de Man, Rogosa, Sharpe) media (Supplementary Table 1) or in an anaerobic medium described elsewhere for aerobic and anaerobic strains respectively (Gervason et al., 2024). After 24 h of culture, 3 % of PC1 was inoculated into fresh media for another 24 h (preculture 2 = PC2). The culture parameters (pH, absorbance, and dry matter) for both precultures were examined to track bacterial development. Following another 24 h of culture, the prebiotic media were inoculated with 5 % of PC2. Prebiotic media consisted of TS, MRS or anaerobic medium depleted in sugars with the plant extract replacing water and sugars in the medium (Fig. 1).

After 40 h of growth, the cultures were centrifuged at 10,000 g for 30 min at 4 °C (Sorvall LYNX 6000, Thermo Scientific, Waltham, MA, USA). The supernatants were filtered at 0.45 μm (Minisart NML, Sartorius, Göttingen, Germany) and mixed with 8 % pasteurized maltodextrin (Glucidex R17, Roquette, Lestrem, France). The bacterial pellets were resuspended in 10 mL of sterile deionized water and inactivated in an oven 1 h at 100 °C (BF 56, Binder, Tuttlingen, Germany). Finally, the inactivated bacteria and the filtered supernatants were mixed together and lyophilized (Cryotec, Lunel-Viel, France) (Fig. 1).

a) Preparation of common chicory extract

The roots of common chicory (*Cichorium intybus*) (France) were cutted into 0.5–2 cm pieces after being dried. Then, 200 g of chicory were extracted in 2000 mL of boiling water and left during 12 h at room temperature. Filtration of the extract was carried out on a 15 μm filter. The extract was concentrated with a rotary evaporator (at 40 °C) to 9.24% of dry matter (dm), containing 61.6%/dm of sugars (analyzed under the monosaccharide form after hydrolysis).

b) Preparation of horse chestnut extract

The seeds of the horse chestnut (*Aesculus hippocastanum*) (Ukraine) were ground into 0.5–2 cm pieces after being dried. Then, 200 g of seeds were extracted in 2000 mL of boiling water and left for 12 h at room temperature. The extract was filtered through a 15 μm filter and concentrated using a rotary evaporator (at 40 °C) to 5.21% dry matter (dm), containing 65.8%/dm of sugars (analyzed in monosaccharide form after hydrolysis).

### 2.2 Structures of bacteria under the scanning electron microscope

Culture and centrifugation of *Akkermansia muciniphila* were performed as previously reported. The bacterial pellets were resuspended in 10 mL of sterile deionized water and inactivated in an oven 1 h at 100 °C (BF 56, Binder). For scanning electron microscopy, bacteria were deposited on polycarbonate filters (0.2 μm) and fixed overnight at 4 °C with 2.5 % glutaraldehyde and paraformaldehyde 2 % in 0.2 M sodium cacodylate buffer, pH 7.4. Bacteria were washed in sodium cacodylate buffer (0.2 mol/L, pH 7.4) and post-fixed 1 h with 1 % osmium tetroxide in same buffer. After rinsing for 20 min in distilled water, dehydration by graded ethanol was performed from 25° to 100° (10 min each) to finish in hexamethyldisilazane for 10 min. Filters were mounted on stubs using adhesive carbon tabs and samples were coated with platinum (Quorum Q150 TES). Observations were carried out using a Field Emission Scanning Electron Microscope Regulus 8230 (Hitachi, Japan) at 2 kV with a secondary electron detector.

### 2.3 Adipose cells

According to the Helsinki Declaration, preadipocyte cells were obtained from anonymous healthy donors from individuals having cosmetic surgery who had no underlying disease. Surgical residue was gathered in compliance with French rules, which included submitting a statement to the Research Ministry (DC no. 2008162) and obtaining written informed permission from patients. Strains were isolated from normal-weighted (Body mass index (BMI) < 20) or obese women (BMI>30). For the differentiation of preadipocytes (PA) into mature adipocytes (MA), cells were seeded at confluence (33,500 cells/cm^2^) in a differentiation medium consisting of Dulbecco's modified Eagle medium (DMEM/F12 (1:1), Gibco, ThermoFisher Scientific, Carlsbad, USA) supplemented with fetal bovine serum (FBS, 10%, Eurobio Scientific, Saclay, France), Gln (1 %), dexamethazon (980 mg/mL), T3 (6.5 mg/mL), hydrocortisone (25 mg/mL), insulin (3.5 mg/mL), rosiglitazone (1.78 mg/mL), isobutyl-methylxanthine (IBMX) (100 mg/mL, only for the first 3 days), and gentamycin (50 mg/mL) (Sigma-Aldrich, St. Louis, MO, USA). The medium was replaced every two days. MAs were obtained after eight days of differentiation.

### 2.4 Generation of adipospheroid

To create an agarose mold, 20 g/L agarose (ThermoFisher Scientific) was mixed with 0.9% w/v NaCl (Sigma-Aldrich), sterilized for 20 min at 120 °C, and placed in MicroTissues® 3D Petri Dishes® (81 wells, Sigma-Aldrich) according to the manufacturer's instructions (Habanjar et al., 2023). In these molds, cell attachment is hindered, and cells spontaneously aggregate to form spheroids by promoting intercellular adhesion molecules. Next, 200,000 preadipocytes/agarose mold were plated and cultivated in DMEM/F12 media (supplemented with 10% FBS, 1% Gln) at 37 °C in 5% CO_2_, resulting in the creation of 81 potential adipospheroids per agarose mold. On the second day, the differentiation medium was applied as described above in order to obtain spheroids made of mature adipocytes called mature adipospheroids.

### 2.5 Blood leukocyte preparation

Blood was collected from healthy human volunteers (EFS, Clermont-Ferrand, France). Donors provided written informed permission for the use of blood samples for research purposes under EFS contract no. 16-21-62 (articles L1222–1, L1222–8, L1243-4, and R1243-61 of the French Public Health Code). Whole blood leukocytes were obtained after hemolytic shock using ammonium chloride solution (NH_4_Cl 115 μM; NaHCO_3_ 12 μM, EDTA 0.01 μM), followed by a centrifugation (1300 rpm, 10 min). Then, they were washed and suspended in Roswell Park Memorial Institute 1640 Medium (RPMI-1640, Gibco, ThermoFisher Scientific), supplemented with FBS (10%), gentamicin (50 μg/mL), and Gln (2 mM).

### 2.6 Peripheral blood mononuclear cells (PBMCs) preparation from human blood

Blood buffy coats were obtained from three healthy human volunteers (EFS, Clermont-Ferrand, France) and carefully layered on a gradient of Ficoll-Histopaque® 1077 (Sigma-Aldrich). The first layer of plasma was aspirated following centrifugation (1500 rpm, 40 min at 25 °C) exhibiting a phase of monocytes and lymphocytes (peripheral blood mononuclear cells, PBMCs) immediately above the 1.077 g/mL layer. The PBMC phase was collected, and the remaining erythrocytes were eliminated using an ammonium chloride solution by hemolytic shock. After centrifugation (1500 rpm, 5 min at 25 °C), the pellet was washed with RPMI, centrifuged twice, and suspended in 5 mL of supplemented RPMI (FBS 10%, gentamicin 50 μg/mL, and Gln 2 mM). The PBMC preparation was then adjusted to 1.10^6^ cells/mL for further assays.

### 2.7 Human monocytic leukemia cells

The human monocytic leukemia cell line THP-1 (American Type Culture Collection ATCC, TIB-202™, Manassas, USA) was cultured and propagated at 37 °C in a humidified atmosphere of 5% CO_2_ in RPMI supplemented with 10% FBS, 2 mM Gln, and 50 μg/mL gentamicin. THP-1 cells (4.10^5^ cells/mL) were incubated in 6-well plates in complete growth medium containing 16.2 nM phorbol 12-myristate 13-acetate (PMA, Sigma-Aldrich) for three days for activation into macrophages. Then, they were polarized into M1-like macrophages by incubating with 10 pg/mL of lipopolysaccharides (LPS) from *Escherichia coli* O26:B6 (Product number: L2654, Sigma-Aldrich) and 20 ng/mL of IFNγ (Gibco) for 24 h.

### 2.8 ROS production by leukocytes

Leukocyte preparations were obtained as previously described. Cells (10^6^ cells/mL) were placed in 96-well plates, incubated with phyto-postbiotics (50 μg/mL) and dihydrorhodamine 123 (Dhr 123, 1 μM, Sigma-Aldrich), and stimulated (or not) by 1 μM phorbol 12-myristate 13-acetate (PMA) for 120 min. The fluorescence intensity of rhodamine 123, which is the product of Dhr 123 oxidation by ROS, was measured every 5 min for 120 min (excitation/emission: 485/535 nm) using the Tecan Spark® (Männedorf, Switzerland). The results were represented as the proportion of ROS generation by stimulated treated cells against stimulated untreated cells (100%).

### 2.9 Leukocyte viability

A suspension of 10^6^ cells/mL (in RPMI supplemented with FBS 10%, gentamicin 50 μg/mL, and Gln 2 mM) was placed in 96-well plates incubated with phyto-postbiotics (50 μg/mL), PMA (0 or 1 μM), and resazurin (25 μg/mL). Fluorescence (excitation/emission: 544/590 nm) was recorded after 2 h using the Tecan Spark®. The results were represented as the proportion of cell viability of the stimulated treated cells compared to stimulated untreated cells (100%).

$$\left(\frac{\text{Resazurine}\;\text{fluorescence}\;\text{of}\;\left(\text{PMA}-\text{stimulated}\;\text{leucocytes}\;\text{treated}\;\text{with}\;\text{the}\;\text{extract}\;\right)}{\text{Resazurine}\;\text{fluorescence}\;\text{of}\;(\text{PMA}-\text{stimulayted}\;\text{leucocytes}\;\text{untreated}}\right)x\;100$$

### 2.10 Co-culture between macrophages and adipospheroids

A co-culture system between macrophages and adipospheroids was used as described previously. Briefly, THP-1 were seeded at the bottom of the wells (250,000 cells/well) and activated into macrophages by the addition of PMA for 3 days. In parallel, adipospheroids were differentiated in agarose molds as described above, and co-cultured with macrophages, in a mixture of adipocyte differentiation medium and macrophage polarization medium (50/50) in the presence of C-Akm extract (50 μg/mL). After 24 h of incubation, total RNA was extracted, and RT-qPCRs were performed as previously described.

### 2.11 Real-time quantitative PCR (RT-qPCR)

Total RNA was extracted with TRIZOL reagent (Invitrogen, ThermoFisher Scientific). After the evaluation of the quantity and purity (Tecan Spark®), DNase treatment (DNase I Amplification grade, Invitrogen) and cDNA retro-transcription (HighCap cDNA RT Kit RNAse inhib, Invitrogen) were made according to the manufacturer's recommendations. Amplification reaction assays were performed using SYBRGreen PCR Master Mix (Life Technologies, Thermo-Fisher Scientific) and primers designed by PrimerExpress software (ThermoFisher Scientific) (Supplementary Table 2) on a StepOne™ (Life Technologies). The expression of the following genes was measured: *GAPDH, IL1-β, TNF-α, IL-6, IL-8, CXCL10, CD163, IL-10, TGF-β, LEPTIN, ADIPONECTIN* and *HSL*. Genes were considered significantly expressed and their transcript measurable if their corresponding Ct value was less than 35. Each sample was normalized to endogenous reference gene (*GADPH*). The relative quantification method (RQ = 2^–ΔΔCt^) was used to calculate the relative gene expression of given samples with ΔΔCt = [ΔCt (sample1) −ΔCT (sample2)] and ΔCt = [Ct(target gene) – Ct(reference gene)].

### 2.12 Determination of cytokine concentrations

PBMCs (10^6^ cells/mL) (4 volunteers) were cultured with or without LPS (10 μg/mL) and C-Akm extract (50 μg/mL) for 24 h. The supernatants were collected and cytokine secretions were measured using the Human Custom ProcartaPlex assays (Invitrogen™; ThermoFisher Scientific). All samples were tested in duplicate for 9 human cytokines/adipokines (IFNγ, IL-2, IL-1β, IL-17A, IL-6, IL-8, TNF-α, Adiponectin, and Leptin). Cytokine levels were measured using the manufacturer's recommended standards and antibody concentrations. Plates were read using the Luminex Bio-Plex 200 system (Biorad, Marnes-la-Coquette, France) and analyzed using BioPlex Manager™ 4.1 software with a five-parameter logistic regression (5 PL) curve fitting. The results were expressed as a percentage of stimulated treated cells relative to untreated stimulated cells (100%).

### 2.13 Untargeted metabolomics

A 50 mg aliquot of C-Akm extract was dissolved in 1 mL of 10 mM ammonium formate. Samples were then centrifuged for 10 min at 10,000 rpm. 200 μL of the supernatant was diluted 2 fold with 200 μL of 10 mM ammonium formate, introduced on a 2 kDa cut off filter (Vivacon 500, SARTORIUS, Göttingen, Germany), and centrifuged for 30 min at 13,000 rpm. The resulting solution was diluted 10 fold with mobile phase A (see below, polarity dependent). A 20 μL aliquot was then injected. For the LC-MS analysis, separation was carried out on an Ultimate 3000 RSLC system (ThermoFisher Scientific) with a ACQUITY BEH C18 100 × 2.1 mm 1.7 μm column (Waters) and the following mobile phases (400 μL/min): for positive mode analysis A: water 0.1% formic acid; B: acetonitrile 0.1% formic acid and for negative mode analysis A: 10 mM ammonium acetate; B: acetonitrile. Gradient separation was 0–1 min, A: 95%, B: 5%; 1–16 min, A: 5%, B: 95%; 16–18 min, A: 5%, B: 95%. Detection (100–1000 Da) was ensured by an Orbitrap Fusion Lumos (ThermoFisher Scientific) high resolution mass spectrometer operated in negative and positive mode (separately, one sequence per mode). The AcquireX mode was used for acquisition, which includes for each sample one injection for precursor mass detection and three injections for iterative precursor fragmentation. For precursor mass detection, a Full MS at R = 240,000 was used. For iterative precursor fragmentation, a FullMS ddMS2 acquisition strategy was used, with ddMS2 scans at R = 30,000. Data processing was carried out with Compound Discoverer 3.3 (ThermoFisher Scientific). Detected compounds (retention time tolerance was 0.7 min) were searched against the mzCloud database considering both their exact mass and fragmentation data for candidate ranking. A match score is calculated based on similarity between experimental fragmentation data and library data; the ChemSpider database considering the formula predicted by the software. Formula prediction was performed considering a 1 ppm mass tolerance together with isotopic pattern detection. Compounds matching the formulae were then ranked by comparing the fragmentation data and a fragment prediction generated by the software, which result in the calculation of a FISh score (Fragment ion Search, score out of 100); the Natural Product Atlas database, filtered for gender “bacterium”. Three different chicory extracts were fermented to produce three distinct C-Akm extracts (n = 3). Metabolomic analysis was conducted on each extract, and the data presented are the average of these three biological replicates.

### 2.14 Statistical analysis

All the experiments were performed 3–10 times. Values are shown as mean ± SEM. Statistical significance between two groups was evaluated using Student's t-test and between more than two groups was evaluated with a one-way ANOVA followed by Tukey's post hoc test using in GraphPad Prism software version 8 (GraphPad Software, San Diego, CA, USA). p < 0.05 was considered significant. Heatmap was plotted with Heatmapper.

## 3 Results

### 3.1 Impact of phyto-postbiotic extracts on ROS production in human leukocytes

**Fig. 2:**
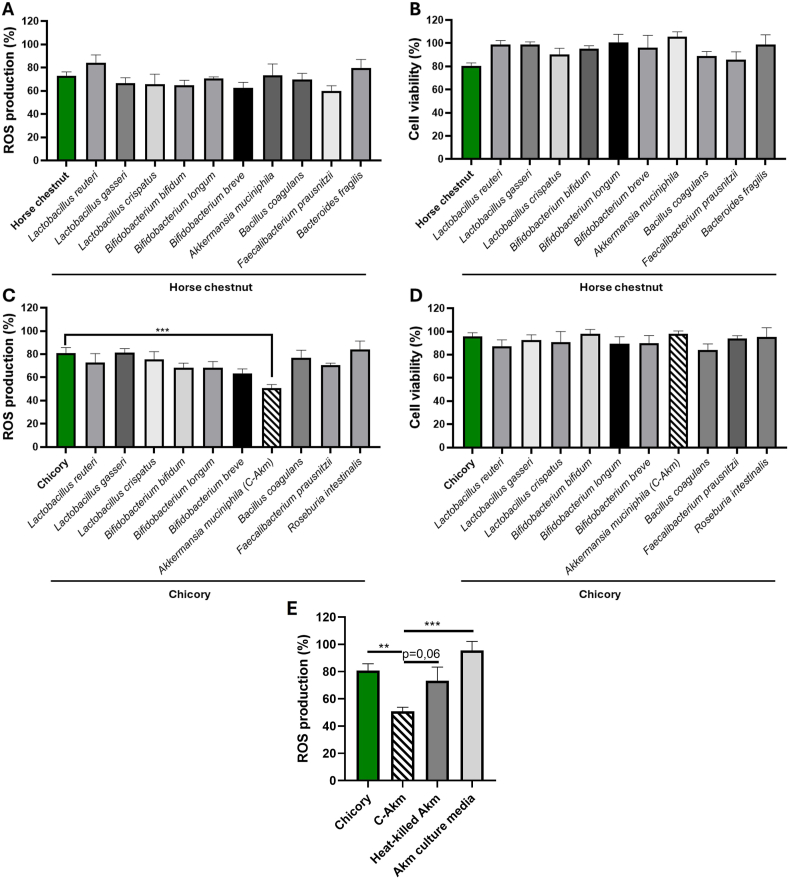
**Effects of phyto-postbiotics extracts on ROS production by blood stimulated-leukocytes and their viability.** Cells were incubated with postbiotics or the control prebiotic (50 μg/mL) and stimulated with PMA (1 μM). After 2h, we measured ROS production with (A) Horse Chestnut combinations and (C) Chicory combinations and (B, D) cell viability by resazurin test. Data are expressed as mean ± SEM (Control = 100 %) and analyzed using Student's t-test (n = 6–10) (versus Horse Chestnut or Chicory), (E) heat-killed Akm and Akm culture media (fermented media without cells) effect on ROS production, data are analyzed using one-way ANOVA followed by Tukey's multiple comparisons test. Differences were considered significant at p < 0.05. ∗p < 0.05, ∗∗p < 0.01, ∗∗∗p < 0.001. Akm: *Akkermansia muciniphila,* PMA: Phorbol myristate acetate, ROS: Reactive oxygen species.

To investigate the potential antioxidant effect of various phyto-postbiotics corresponding to the combination of two potential prebiotics fermented by ten potential probiotics, we examined their effect on blood leukocyte ROS production triggered by PMA. We wanted to study if our combinations had a stronger antioxidant effect than prebiotics alone. All the extracts produced by the fermentation with horse chestnut reduced ROS production (approximately 20% reduction for the least active at 50 μg/mL) (Fig. 2A) without affecting cell viability (Fig. 2B). However, none of them showed a significantly greater reduction than horse chestnut alone (−28 ± 8% at 50 μg/mL). Extracts fermented with chicory also reduced ROS production (around 20% for the least active), and the combination of chicory and *Akkermansia muciniphila* (called C-Akm) produced a greater reduction than chicory alone (−49 ± 4% *vs* −19 ± 7%, p < 0.001) (Fig. 2C). This effect was not the consequence of a decrease in cell viability (Fig. 2D). As well as examining the impact of our prebiotic alone and in combination, we wanted to investigate how the probiotic and culture supernatant from our most active combination affected ROS generation. Results showed that C-Akm extract outperformed heat-inactivated *Akkermansia muciniphila,* although not significantly (−49 ± 4% *vs* −26 ± 13%, p < 0.06) and the culture supernatant which contains bacterial metabolites (−49 ± 4% *vs* −5±8%, p < 0.001) (Fig. 2E). For the remainder of the study, we decided to retain the C-Akm extract, which was the most active.

### 3.2 Heat treatment did not induce an alteration in morphology or size of *Akkermansia muciniphila*

**Fig. 3:**
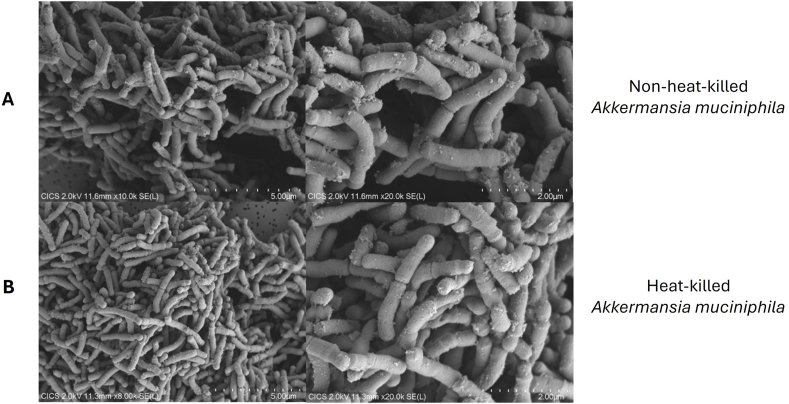
**Impact of heat inactivation on *Akkermansia muciniphila* morphology.** Scanning electron microscopy images of (A) Non-heat-killed *Akkermansia muciniphila*, (B) Heat-killed *Akkermansia muciniphila.*

The effects of heat inactivation on the morphology of *Akkermansia muciniphila* were studied using scanning electron microscopy. We found no difference in morphology or size between non-heat-killed and heat-killed *Akkermansia muciniphila* (Fig. 3). Heat-killed bacteria were then subcultured in liquid medium to check for inactivation. No bacterial growth was detected (data not shown).

### 3.3 Metabolite identification in C-Akm extract

**Fig. 4:**
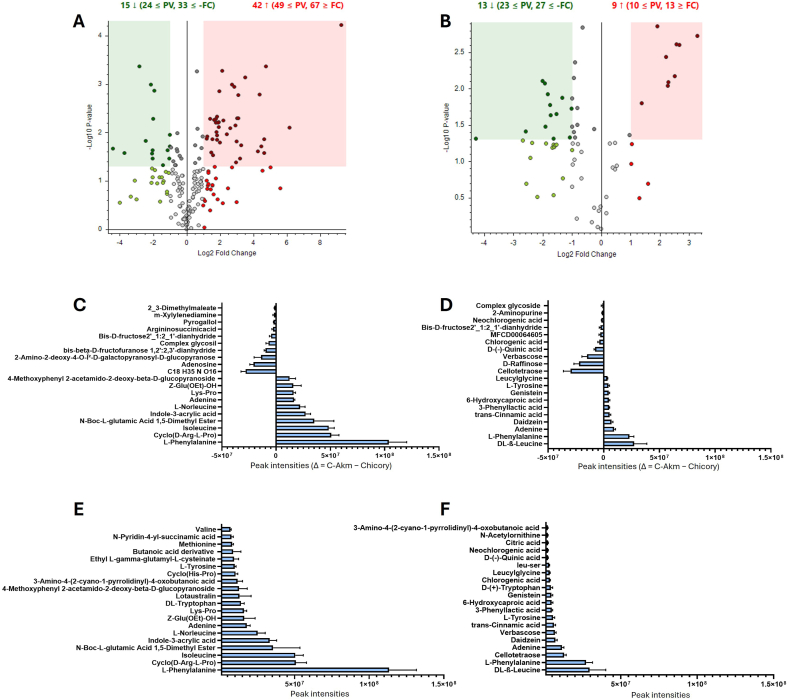
**Untargeted metabolomics**. Volcano plots showing the comparison of chicory fermented by *Akkermansia muciniphila* extract (C-Akm) and Chicory extracts in the positive (A) and negative (B) ion modes. The differential presence of the metabolites was determined as fold-change ratio (C-Akm extract/Chicory extract, >1 or < −1, p < 0.05 multivariate paired *t*-test). Each point represents a metabolite. Red dots represent upregulated metabolites, and green dots represent downregulated ones. The metabolite peak intensity between the C-Akm extract and the chicory extract, expressed as delta (Δ = C-Akm - Chicory) in positive (C) and negative (D) ion modes, represents which metabolites were primarily generated and which were consumed. Top 20 metabolites with the highest peak intensity in C-Akm extract in the positive (E) and negative (F) ion modes. C-Akm: *Akkermansia muciniphila* extract.

In order to determine which metabolites were found differentially in the C-Akm and Chicory groups, an untargeted metabolomic analysis was carried out using positive and negative ionizations. The volcano plots in Fig. 4A and B showed the global distribution of the numerous metabolites. The differential expression was determined as fold-change ratio (C-Akm extract over Chicory extract) (>1 or < -1, p < 0.05 multivariate paired *t*-test). In the positive ionization assay (Fig. 4A), 57 metabolites were differentially found, 15 were down-regulated, and 42 up-regulated. The negative ionization study (Fig. 4B) revealed that 22 metabolites were differentially found, 13 down-regulated, and 9 up-regulated. However, this approach is based on ratios, and many metabolites could be either completely consumed or simply created. In Fig. 4C and D, we used the difference between the metabolites found after fermentation and before fermentation (Δ = C-Akm extract – Chicory extract) to determine which metabolites were primarily generated and which one were consumed. We can observe that the main metabolites generated are amino acids and acids (L-phenylalanine, isoleucine, adenine) (Fig. 4C and D). We also noticed the synthesis of daidzein and genistein (Fig. 4D). As we can see in Fig. 4C and D, sugars are the main compounds used by the bacteria. Indeed, many sugars (verbascose, raffinose, cellotetraose, glucopyranose, pyrogallol) have a strong negative intensity, indicating that they were more present before fermentation. To characterize the C-Akm extract, we have compiled a list of 20 compounds with the most intense peak (Fig. 4E and F) and we showed that the C-Akm extract contained a variety of amino acids (BCAA, aromatic amino acids), polyphenols, and some acids (L-phenylalanine, Cyclo(D-Arginine L-Proline), isoleucine, indole-3-acrylic acid, daidzein). We also used HPLC to determine if the fermentation induced the synthesis of SCFAs, and we detected the production of acetic acid (6.5 g/L) but no butyric or propionic acid. Additionally, lactic acid and citric acid were generated (8.6 g/L and 3.2 g/L, respectively) (Supplementary Table 3).

### 3.4 C-Akm extract modulated gene expression implicated in macrophage polarization

**Fig. 5:**
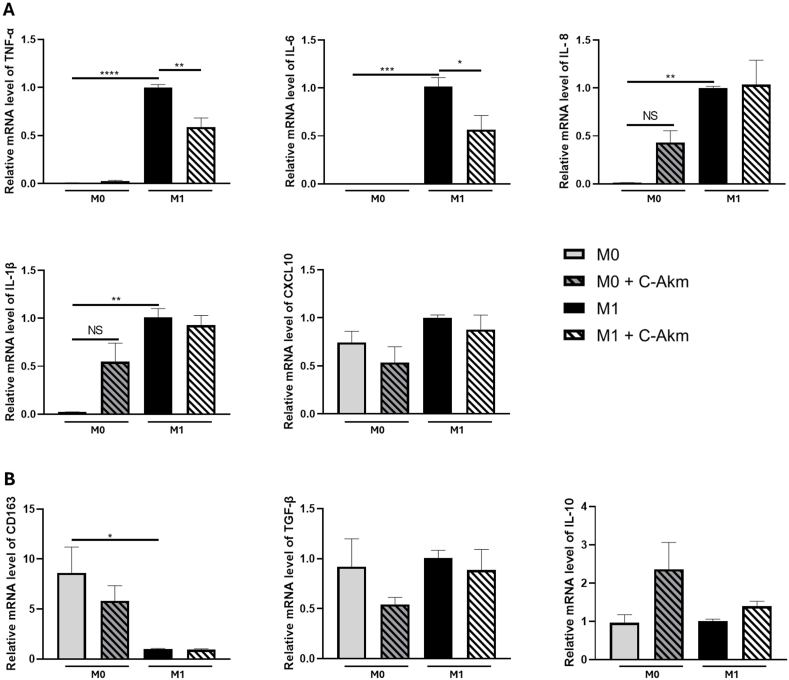
**Effects of C-Akm extract on macrophage polarization.** THP-1 cells were activated with PMA (10 μM) and then with LPS and IFNy to polarize into M1-type. Treatment with C-Akm (50 μg/mL) was investigated at each step. Gene expression was quantified by real-time qPCR and normalized using GAPDH as an internal control. (A) M1 macrophages markers (B) M2 macrophages markers. Data were expressed as mean ± SEM and analyzed using one-way ANOVA followed by Tukey's multiple comparisons test (n = 3). Differences were considered significant at p < 0.05. ∗p < 0.05, ∗∗p < 0.01, ∗∗∗p < 0.001, ∗∗∗∗p < 0.0001 C-Akm: chicory fermented by *Akkermansia muciniphila*, CD163: Cluster of differentiation 163, CXCL10: Human C-X-X motif chemokine ligand 10, GADPH: Glyceraldhehyde 3-phosphate dehydrogenase, IFNy: Interferon gamma, IL-1β: Interleukine 1-beta, IL-6: Interleukine-6, IL-8: Interleukine-8, IL-10: Interleukine-10, LPS: Lipopolysaccharides, PMA: Phorbol myristate acetate, TGF-β: Transforming growth factor beta, TNF-α: Tumor necrosis factor alpha.

As macrophages are crucial actors in the link between inflammation and obesity, the impact of C-Akm extract (50 μg/mL) was assessed on THP-1 cells activated into macrophages M0-like and during the polarization stage for the formation of pro-inflammatory M1-like macrophages. At baseline (M0), C-Akm extract did not significantly increase the expression of the studied genes (TNF-α, IL-6, IL-8, IL-1β, CXCL10) (Fig. 5A). When we assessed the impact of C-Akm extract during the polarization of macrophages into M1-type, C-Akm extract significantly decreased the expression of *TNF-α* (RQ = 0.58 ± 0.11, p < 0.01) and *IL-6* (RQ = 0.56 ± 0.18, p < 0.05). The extract did not significantly affect the expression of *IL-8* (RQ = 1.03 ± 0.32), *IL-1β* (RQ = 0.92 ± 0.13), and *CXCL10* (RQ = 0.87 ± 0.18). Then, after observing a decrease in gene expression of M1 markers (*TNF-α* and *IL-6*), we investigated if C-Akm extract could polarize these macrophages into M2-like (Fig. 5B). C-Akm extract did not significantly raise the expression of two M2 gene markers studied (*CD163* and *TGF-β*) in both the M0 and M1 conditions. Concerning *IL-10*, C-Akm extract induced a non-significant increase in IL-10 expression in both basal (RQ = 2.35 ± 0.88 vs RQ = 0.95 ± 0.25) and M1 conditions (RQ = 1.40 ± 0.17).

### 3.5 C-Akm extract significantly decreased the production of pro-inflammatory cytokines in LPS-stimulated PBMCs

**Fig. 6:**
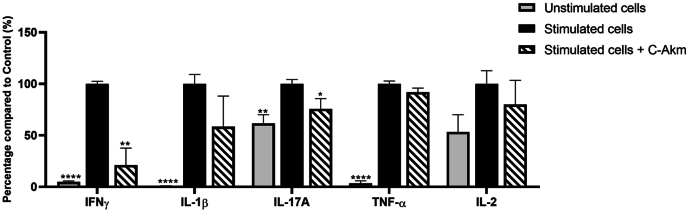
**Effects of C-Akm extract on LPS-stimulated PBMCs**. Cells were incubated with or without LPS (10 μg/mL) and C-Akm (0 or 50 μg/mL) for 24 h. Cytokine levels were measured with the Luminex Bio-Plex 200 System using optimal concentrations of standards and antibodies according to the manufacturer's instructions. Data are expressed as mean ± SEM (Stimulated cells = 100%) and analyzed using Student's t-test (n = 4) (versus Stimulated cells 100 %). Differences were considered significant at p < 0.05. ∗p < 0.05, ∗∗p < 0.01, ∗∗∗∗p < 0.0001. C-Akm: chicory fermented by *Akkermansia muciniphila*, IFNy: Interferon gamma, IL-1β: Interleukine 1-beta, IL-2: Interleukine-2, LPS: Lipopolysaccharides, PBMCs: Peripheral blood mononuclear cells, TNF-α: Tumor necrosis factor alpha.

To investigate the impact of C-Akm extract on cytokine production, LPS-stimulated and non-LPS-stimulated PBMCs were treated with 50 μg/mL C-Akm. The secretion of 5 cytokines was measured in cell supernatants using the Luminex Bio-Plex 200 system (Fig. 6). C-Akm extract treatment significantly reduced IFNγ (−79 ± 22%, p < 0.01) and IL-17A levels (−24 ± 16%, p < 0.05) compared to stimulated non-treated cells. The treatment with C-Akm extract reduced IL-1β and IL-2 but non-significantly (−58 ± 61%, −20 ± 28% respectively), and did not affect TNF-α release by PBMCs.

### 3.6 C-Akm extract affected the expression and secretion of cytokines by human mature adipospheroids

**Fig. 7:**
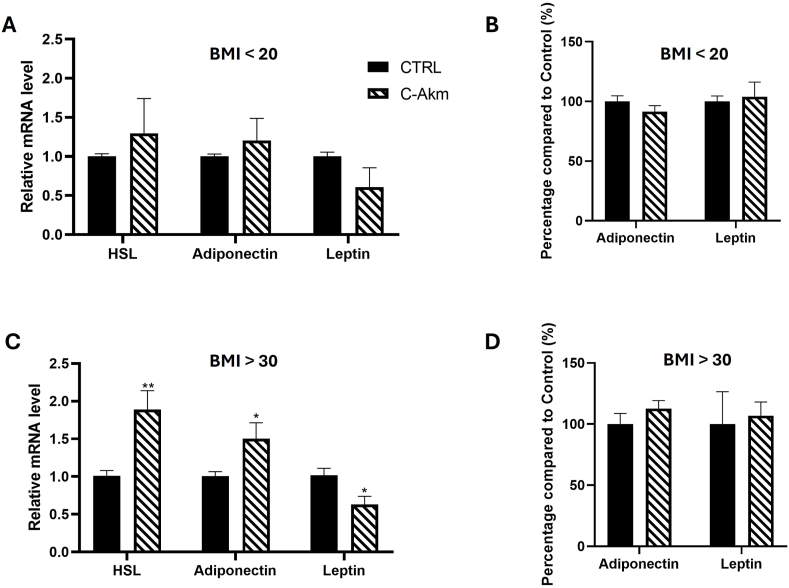
**Effect of C-Akm extract on adipokine expression in human mature adipospheroids.** Mature adipospheroids were treated with 50 μg/mL of C-Akm for 24 h. Total RNA isolated from untreated cells was used as the control. Data showed the relative mRNA expression of Leptin, Adiponectin and HSL normalized to GAPDH. (A) Leptin, adiponectin and HSL expression on mature adipospheroids from normal weighted women (BMI<20), (B) Leptin and adiponectin secretions by mature adipospheroids (BMI<20) were measured with the Luminex Bio-Plex 200 System using optimal concentrations of standards and antibodies according to the manufacturer's instructions (Control = 100 %), (C) Leptin, Adiponectin and HSL expression in mature adipospheroids from obese women (BMI>30) (D) Leptin and adiponectin secretions by mature adipospheroids (BMI>30) were measured with the Luminex Bio-Plex 200 System (Control = 100 %). Data were expressed as mean ± SEM and analyzed using Student's t-test (n = 5) (vs CTRL). Differences were considered significant at p < 0.05. ∗p < 0.05, ∗∗p < 0.01. BMI: Body mass index, C-Akm: chicory fermented by *Akkermansia muciniphila*, GAPDH: Glyceraldhehyde 3-phosphate dehydrogenase, HSL: Hormone-sensitive lipase.

The impact of the C-Akm extract was then evaluated on human mature adipospheroids constituted of adipose cells from normal-weighted women (BMI<20) or obese women (BMI>30). Human preadipocytes were used to generate spheroids, which were differentiated as explained above for 8 days. Mature adipospheroids were treated with C-Akm extract (50 μg/mL) extract for 24 h to evaluate adipokine and *HSL* (hormone-sensitive lipase) gene expression. C-Akm extract did not have any significant effect concerning *Leptin, Adiponectin* and *HSL* expression in adipospheroids derived from preadipocytes of normal-weighted individuals (BMI<20) (Fig. 7A). The secretion of leptin and adiponectin remained unaffected by the treatment. (Fig. 7B). On the contrary, C-Akm extract significantly decreased *Leptin* expression (RQ = 0.63 ± 0.17, p < 0.05) and increased *Adiponectin* (RQ = 1.50 ± 0.27, p < 0.05) and *HSL* expression (RQ = 1.88 ± 0.39, p < 0.01) compared to untreated controls in adipospheroids derived from preadipocytes of obese people (Fig. 7C). Leptin and adiponectin secretion remained unchanged following treatment (Fig. 7D).

### 3.7 Effect of C-Akm extract on the interaction between adipocyte/macrophage

**Fig. 8:**
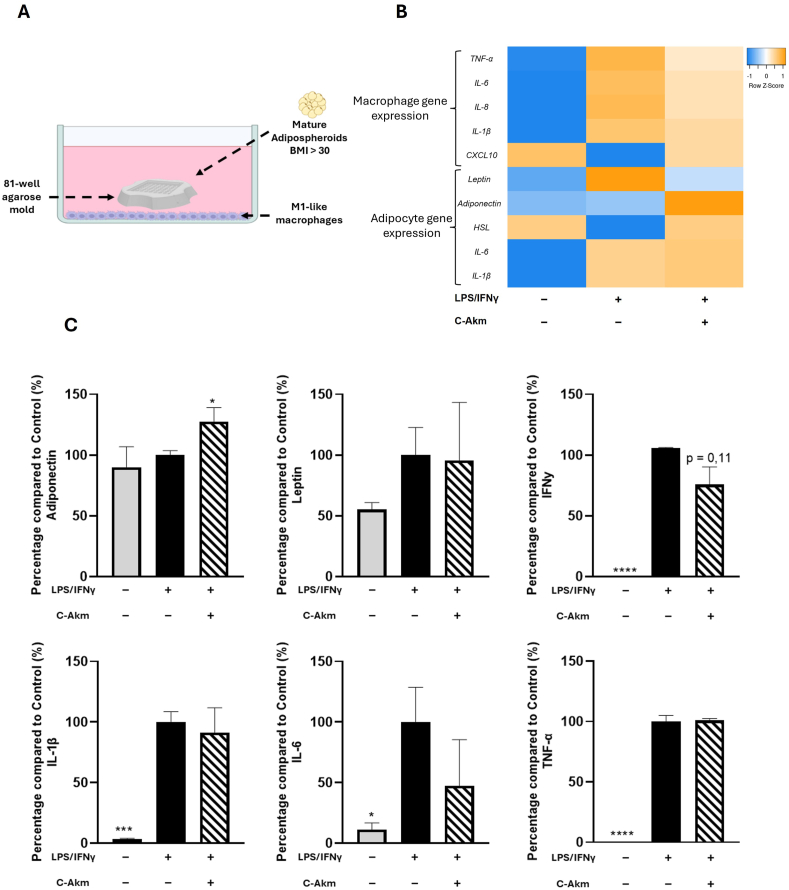
**Effets of C-Akm on Adipocyte/Macrophage Interactions.** (A) Graphical representation of the co-culture system in which M1-like proinflammatory macrophages (in the basal part of the well) were co-cultured with mature adipospheroids in agarose molds; (B) Heatmap representing mRNA expression of *IL-6, TNFα, IL-1β, IL-8* and *CXCL10* by M1 cells and *Leptin*, *Adiponectin*, *HSL*, *IL-1β*, *IL-*6 by human mature obese adipospheroids after 24 h of co-culture measured by qRT-PCR. Total RNA isolated from untreated M1 macrophages or adipospheroids was used as the control. Normalized expression values are z score normalized for each gene (C). The secretion of 6 cytokines was measured with the Luminex Bio-Plex 200 System using optimal concentrations of standards and antibodies according to the manufacturer's instructions. Data were expressed as mean ± SEM (Control = 100%) and analyzed using Student's t-test (n = 4) (vs CTRL). Differences were considered significant at p < 0.05. ∗p < 0.05, ∗∗∗p < 0.001, ∗∗∗∗p < 0.0001. BMI: Body mass index, C-Akm: chicory fermented by *Akkermansia muciniphila*, CXCL10: Human C-X-X motif chemokine ligand 10, HSL: Hormone-sensitive lipase, IFNy: Interferon gamma, IL-1β: Interleukine 1-beta, IL-6: Interleukine-6, IL-8: Interleukine-8, LPS: Lipopolysaccharides, TNF-α: Tumor necrosis factor alpha.

To simulate an inflamed obese adipose tissue, we realized a co-culture system between M1-like pro-inflammatory macrophages (at the bottom of the wells) and human mature adipospheroids constituted by adipose cells from an obese patient differentiated in agarose mold as described previously (Fig. 8A). This model permitted to investigate the effect of C-Akm extract on the inflammatory state generated by the interaction between M1 macrophages and adipocytes. The differential gene expression of the two cell types was presented as a heatmap (Fig. 8B). In macrophages, C-Akm extract significantly reduced *TNF-α* (RQ = 0.72 ± 0.14, p < 0.01), *IL-6* (RQ = 0.78 ± 0.06 p < 0.01), and *IL-8* (RQ = 0.77 ± 0.07 p < 0.01). C-Akm extract had no effect on *CXCL10* and *IL-1β* expression. In adipospheroids, C-Akm extract significantly decreased the expression of *Leptin* (RQ = 0.80 ± 0.04, p < 0.01), increased that of *Adiponectin* and *HSL* (RQ = 2.09 ± 0.66 p < 0.05 and RQ = 1.89 ± 0.61 p < 0.01 respectively) and had no impact on *IL-6* or *IL-1β* expression levels. Concerning secretions (Fig. 8C), C-Akm extract significantly increased adiponectin secretion (+27 ± 15%, p < 0.05) and lowered IL-6 (−52 ± 50%) and IFNy (−25 ± 19%) secretions but not significantly.

## 4 Discussion

Obesity is regarded as a major public health issue and receives significant attention from the WHO and governments around the world, with prevalence rising steadily (Lingvay et al., 2024). Obesity is characterized by hyperplasia and hypertrophy of adipocytes in adipose tissue, as well as a polarization of immune cells, which promote inflammation (Longo et al., 2019). Ultimately, this chronic low-grade inflammation contributes to the development of numerous diseases (Pi-Sunyer, 2009). Current anti-obesity medications primarily target appetite, acting on mechanisms of satiety, such as GLP-1 analogs (Gudzune and Kushner, 2024). However, they do not address the inflammation associated with obesity. Fermented foods, probiotics, and postbiotics offer promising prospects in the fight against obesity and inflammation (Vázquez-Cabral et al., 2017; Yong et al., 2019; Wu et al., 2021; Wang et al., 2018). For instance, the gut bacteria *Akkermansia muciniphila* has been associated with improved metabolic health and reduced inflammation (Xu et al., 2020; Schneeberger et al., 2015). Furthermore, the first clinical proof of concept was demonstrated in a study where supplementation with either live or pasteurized *Akkermansia muciniphila* at a dose of 10^10^ CFU/day for 3 months significantly improved insulin sensitivity, decreased plasma insulin levels and total cholesterol, and slightly reduced body weight compared to the placebo group (Depommier et al., 2019).

In our investigation, we first focused on the production of fermented extracts before investigating their impact on the main actors of obesity responsible for chronic inflammation: adipocytes and macrophages. We began with the hypothesis that fermenting prebiotics with probiotic microorganisms would produce secondary metabolites beneficial to health (SCFA, amino acids, vitamins). Our study revealed that fermentation led to the generation of acetate, but not butyrate or propionate. This aligns with the established function of *Akkermansia muciniphila* in the production of short-chain fatty acids, chiefly acetate and propionate, *via* mucin breakdown (Derrien et al., 2004). Additionally, we detected amino acids, polyphenols, and organic acids. Furthermore, heat-inactivated probiotic bacteria have already been proven to have anti-inflammatory, antioxidant, and anti-obesity properties (Uchinaka et al., 2018; Sun et al., 2023; Jang et al., 2023). We demonstrated that our thermal inactivation at 100 °C did not appear to impair either the morphology or the size of *Akkermansia muciniphila*, which is consistent with previous studies on probiotic bacteria (Xu et al., 2023; Ali et al., 2022). Although the shape is oval, it has been shown previously to be rather elongated using scanning electron microscopy, which might be explained by hydrophobicity, self-aggregation, and, in our study, post-culture centrifugation (Wu et al., 2023a; Yin et al., 2021).

In obese people, mitochondrial malfunction has been seen in adipocytes in adipose tissue, resulting in a diminished ability to oxidize fatty acids and an increased ROS generation (de Mello et al., 2018; Woo et al., 2019). Furthermore, obesity induces metabolic stress which contributes to the production of ROS (Furukawa et al., 2017). This production of ROS plays a central role in the progression of inflammatory diseases. They act as both signaling molecule and inflammatory mediator (Forrester et al., 2018). Numerous *in vitro* and *in vivo* investigations have found that certain probiotic bacteria, mostly *Lactobacillus* species, possess antioxidant properties (Vougiouklaki et al., 2023; Amaretti et al., 2013; Singh et al., 2017). However, there have been few investigations on the antioxidant properties of *Akkermansia muciniphila*. Wu and collaborators found that treating *Caenorhabditis elegans* with a pasteurized *Akkermansia muciniphila* extract reduced ROS accumulation while increasing SOD and GSH-Px activity (Wu et al., 2022). Our results suggested that C-Akm extract treatment reduced ROS generation in PMA-stimulated leukocytes. Furthermore, we found that heat-killed Akm and Akm culture media (fermented media without cells) only resulted in a slight decrease in ROS generation. Inactivated *Akkermansia muciniphila* and the metabolites generated by the fermentation therefore appeared to act synergistically. The oxidative environment of adipose tissue promotes the infiltration and activation of M1 macrophages (Russo and Lumeng, 2018). The production of ROS, which is substantially larger in M1-polarized macrophages than in M2, serves as a positive feedback loop. ROS can activate signaling pathways such as nuclear factor kappa B (NF-κB) and mitogen activated protein kinase (MAPK), which regulate M1 macrophage phenotype and function (Canton et al., 2021; Son et al., 2011). A first study showed that pre- and post-treatment of THP-1 derived macrophages with live *Akkermansia muciniphila*, before and after stimulation with gliadin, induced an anti-inflammatory phenotype (M2) associated with a decrease in pro-inflammatory cytokines (IL-6, TNF-α) and an increase in anti-inflammatory cytokines (IL-10, TGF-β) compared to the group treated with gliadin alone (Molaaghaee-Rouzbahani et al., 2023). A second study, conducted *in vivo* on a mouse model of periodontitis caused by *Porphyromonas gingivalis*, found that administering live *Akkermansia muciniphila* or Amuc_1100 significantly increased the population of anti-inflammatory M2 macrophages while decreasing the population of inflammatory M1 macrophages. The same study demonstrated that adding *Akkermansia muciniphila* to bone marrow-derived macrophages infected with *P. gingivalis* boosted IL-10 production while having no effect on TNF-α secretion (Mulhall et al., 2020). Another research yielded opposing results. This last study demonstrated *in vitro* in RAW264.7 that treatment with live *Akkermansia muciniphila* appears to induce a pro-inflammatory response, with an increase in the expression of pro-inflammatory cytokines as well as an increase in the expression of the surface markers CD40 and CD80 (Calvo et al., 2024). Our findings reveal that C-Akm extract reduced TNF-α and IL-6 expression in macrophages that are polarized in M1. C-Akm extract appears to modulate macrophage polarization in an M1 condition. Our investigation found that C-Akm extract reduces the production of IFNγ, IL-17A, and IL-1β on LPS-stimulated PBMCs. However, when compared to previous *in vivo* or *in vitro* research using live or inactivated bacteria, we found no influence on TNF-α release (Zheng et al., 2023a; Shi et al., 2022; Ashrafian et al., 2021). These anti-inflammatory and antioxidant properties might be explained by the effect of C-Akm extract on various inflammation-related pathways such as NF-κB, AMPK, and nuclear factor erythroid 2-related factor 2 (Nrf2). Indeed, several studies have shown *in vivo* and *in vitro* an influence of *Akkermansia muciniphila* on the inactivation of NF-κB, particularly by decreasing its phosphorylation (Shi et al., 2022; Ma et al., 2024; Shen et al., 2023). Furthermore, Shi et al. demonstrated in an *in vitro* study on Caco-2 cells pre-treated with living or pasteurized bacteria and then stimulated with LPS, an increase in the level of phosphorylated AMPK (Shi et al., 2022). In addition to *Akkermansia muciniphila's* activity on these inflammatory components, metabolites produced during fermentation may influence these signaling pathways. Indeed, Lee et al. found that treating LPS-stimulated RAW 264.7 macrophages with branched chain amino acids (BCCA) suppressed NO generation and decreased LPS-induced mRNA expression of IL-6 and cyclooxygenase-2 (COX-2) (Lee et al., 2017). Furthermore, Anan et al. demonstrated that aromatic amino acids and their metabolites reduced IFNy signaling in THP-1 and A549 monocytes challenged with LPS. More broadly, amino acids have been found to have anti-inflammatory properties in the gut, particularly *via* suppressing NF-κB and activating Nrf2 and antioxidant response elements (AREs) (He et al., 2018; Egbujor et al., 2024). Other fermentation-derived metabolites, such as daidzein and genistein, are known to have antioxidant and anti-inflammatory properties by inhibiting the NF-κB and signal transducer and activator of transcription 1 (STAT-1) pathways while activating the AMPK pathway (Hämäläinen et al., 2007; Ji et al., 2012; Choi et al., 2012). Furthermore, other polyphenols present in our extract, such as chlorogenic acid and neochlorogenic acid, have previously been shown to exert anti-inflammatory and antioxidant effects, particularly by acting on Nrf2, AMPK, and NF-κB (Gao et al., 2020; Hwang et al., 2014). Lactic acid, acetic acid and citric acid are also known to have potential roles in anti-inflammatory activities (Peter et al., 2015; Yang et al., 2019; Singh et al., 2022).

Additionally to the inflammatory component associated with immune cells in obese adipose tissue, adipocytes are also immunocompetent cells. In fact, they share receptors with immune cells, such as Toll-like receptor 2 (TLR2), TLR4, tumor necrosis factor receptor (TNFR), and Interleukin 6 receptor (IL-6R), and may secrete cytokines called adipokines (Cawthorn and Sethi, 2008; Bès-Houtmann et al., 2007; Päth et al., 2001). Obesity also causes dysregulation of adipokine production, with increased leptin levels and decreased adiponectin levels. Zhao et al. found that supplementation with live *Akkermansia muciniphila* in mice fed a chow diet reduced plasma leptin levels (Zhao et al., 2017). Another study by Wu et al. showed that *Akkermansia muciniphila* supplementation significantly reduced serum insulin, leptin and resistin levels, while adiponectin was comparable between control and treated groups (Wu et al., 2023b). Plovier et al. reported comparable effects in obese and diabetic mice after supplementing with live *Akkermansia muciniphila*, including a drop in leptin levels. They also discovered a higher reduction in leptin levels in mice treated with pasteurized microorganisms (Plovier et al., 2017). Our study revealed that C-Akm extract administration had no effect on leptin and adiponectin gene expression or protein levels in human mature adipospheroids made from pre-adipocytes from lean people (BMI = 19). In human mature obese adipospheroids (BMI >30), C-Akm extract decreased *leptin* expression while increasing *adiponectin* expression. Although not statistically significant, adiponectin protein levels increased. The extract also increased the expression of *HSL*, an enzyme involved in lipolysis. These findings are consistent with *in vitro* investigations on 3T3-L1 and *in vivo* experiments employing pasteurized *Akkermansia muciniphila* and Amuc_1100 (Depommier et al., n.d.; Zheng et al., 2023b). In the co-culture model mimicking inflamed obese adipose tissue, we observed the same results as during THP-1 polarization into M1, i.e. a decrease in *TNF-α* and *IL-6* expression, as well as a decrease in IL-8. As regards the effect on inflamed mature obese adipocytes, C-Akm extract treatment had the same effect as on human mature obese adipocytes. Furthermore, the C-Akm extract did not affect the expression of pro-inflammatory cytokines on adipocytes. At protein level, this extract increased adiponectin while decreasing IFNγ and IL-6. These findings might be explained by the activity of C-Akm extract, which reduces inflammation as previously demonstrated, but also acts on APMK and PPARγ, which is associated with an increase in adiponectin levels (Daval et al., 2006).

To our knowledge, this is the first study to generate and characterize an extract derived from the fermentation of chicory by *Akkermansia muciniphila*. In addition to using human immune cells, we also used human matures adipocytes organized into adipospheroids, which is a complementary and original *in vitro* tool to investigate deeply in mechanism of action. Indeed, most *in vitro* tests on adipogenesis and anti-obesity effects are performed on 3T3-L1, a murine cell line. One of the significant findings of our study was that C-Akm extract promoted the interaction between adipocytes and macrophages, notably by lowering inflammation. Based on our findings and existing literature, we hypothesize that our extract may exert its effects primarily through the activation of Nrf2, PPARγ, and AMPK, as well as the inhibition of NF-κB.

The fundamental disadvantage of *in vitro* experiments is that the results cannot be replicated in an organism; one of our study's drawbacks is the difficulty of reflecting the results *in vivo*. It would be interesting to do research on high fat-diet mice to determine the effect of the extract on the animal's general physiology, particularly its intestinal microbiota since the composition of our extract may have an impact on the modulation of the intestinal microbiota. Indeed, Minj et al. and other studies have shown that some natural products can prevent diet-induced obesity by modifying the gut microbiota, particularly by increasing the amount of *Akkermansia muciniphila* (Minj et al., 2024). Furthermore, this *in vivo* study could provide deeper insights into the mechanistic workings of our extract on inflammation and obesity. Finally, our research allowed us to generate and characterize a fermented chicory extract produced by *Akkermansia muciniphila*. Furthermore, we demonstrated that C-Akm extract might be a promising option for managing obesity by lowering low-grade inflammation and modulating adipokine production.

## CRediT authorship contribution statement

**A. Chervet:** Investigation, Methodology, Writing – original draft. **R. Nehme:** Investigation, Methodology. **C. Defois-Fraysse:** Investigation, Methodology. **C. Decombat:** Investigation, Methodology. **C. Blavignac:** Investigation, Data curation. **C. Auxenfans:** Methodology. **B. Evrard:** Methodology, Validation. **S. Michel:** Investigation. **E. Filaire:** Project administration, Funding acquisition. **J.-Y. Berthon:** Project administration, Funding acquisition. **A. Dreux-Zigha:** Conceptualization, Methodology. **L. Delort:** Methodology, Investigation, Supervision, Writing – review & editing. **F. Caldefie-Chézet:** Project administration, Funding acquisition, Writing – review & editing.

## Informed consent statement

All donors gave their written informed consent for the use of blood samples for research purposes under Établissement Français du Sang contract no. EFS AURA 22–106 (in accordance with articles L1222–1, L1222–8, L1243–4 and R1243-61 of the French Public Health Code). Human adipose stem cells (hASCs) were provided by the Cell and Tissue Bank (Hôpital Edouard-Herriot, Lyon, France). hASCs were obtained from patients undergoing surgery for cosmetic purposes without associated pathology, in accordance with the Helsinki Declaration, from anonymous healthy donors. Surgical residue was harvested in accordance with French regulation, including declaration to the Research Ministry (DC no. 2008162) and procurement of written informed consent from the patients.

## Funding

This research was funded by Project ANR-19-LCV2-0003-01 Program LABCOM 2019PHYTOPROB’INOV (AV0027085). The project is co-financed by the 10.13039/501100008530European Regional Development Fund (FEDER).

## Declaration of Competing interest

Authors “Clemence Defois-Fraysse, Assia-Dreux Zigha, Jean-Yves Berthon” were employed by the company Greentech/Greencell. The remaining authors declare that the research was conducted in the absence of any commercial or financial relationships that could be construed as a potential conflict of interest.

## Data Availability

Data will be made available on request.
