# Supplementary material for: Development and characterization of a chicory extract fermented by Akkermansia muciniphila: An in vitro study on its potential to modulate obesity-related inflammation
Source: Curr Res Food Sci. 2025 Jan 16;10:100974. doi: 10.1016/j.crfs.2025.100974 (PMC11791162; doi:10.1016/j.crfs.2025.100974)
Supplement: Multimedia component 1 [file mmc1.docx]

**Supplementary table 1: Culture media composition**

| **MRS cosmos media** | **Quantity (g/L)** |
| --- | --- |
| **K2HPO4,3H2O** | 2 |
| **MgSO4,7H2O** | 0.2 |
| **MNSO4,H2O** | 0.05 |
| **Yeast extract** | 15 |
| **Glucose 200g/L** | 10% (100 mL) |

| **TS cosmos media** | **Quantity (g/L)** |
| --- | --- |
| **Yeast extract** | 17 |
| **Soy Peptone** | 3 |
| **NaCl** | 5 |
| **K2HPO4,3H2O** | 2.5 |
| **Glucose** | 2.5 |

**Supplementary table 2:** PCR primer sequences

| **Gene** | **Species** | **Forward Primer sequence (5’-3’)** | **Reverse Primer sequence (5’-3’)** |
| --- | --- | --- | --- |
| **GAPDH** | Human | CACATGGCCTCCAAGGAGTAA | TGAGGGTCTCTCTCTTCCTCTTGT |
| **IL-8** | Human | CTGGCCGTGGCTCTCTTG | CCTTGGCAAAACTGCACCTT |
| **IL-1β** | Human | CCTGTCCTGCGTGTTGAAAGA | GGGAACTGGGCAGACTCAAA |
| **IL-6** | Human | GCTGCAGGCACAGAACCA | ACTCCTTAAAGCTGCGCAGAA |
| **TNF-α** | Human | TCTTCTCGAACCCCGAGTGA | GGAGCTGCCCCTCAGCTT |
| **CXCL10** | Human | GGAAATCGTGCGTGACATTA | AGGAAGGAAGGCTGGAAGAG |
| **TGF-β** | Human | GACATCAAAAGATAACCACTC | TCTATGACAAGTTCAAGCAGA |
| **CD163** | Human | CGGTCTCTGTGATTTGTAACCAG | TACTATGCTTTCCCCATCCATC |
| **IL-10** | Human | GACTTTAAGGGTTACCTGGGTTG | TCACATGCGCCTTGATGTCTG |
| **Leptin** | Human | CGGAGAGTACAGTGAGCCA | CGGAATCTCGCTCTGTCAT |
| **Adiponectin** | Human | CCCAAAGAGGAGAGGAA | TCAGAAACAGGACACAAC |
| **HSL** | Human | GCCTGGGCTTCCAGTTCAC | CCTGTCTCGTTGCGTTTGTAGT |

**Supplementary table 3:** Fermentation products detected by HPLC

|  | **Chicory** | **C-Akm** |
| --- | --- | --- |
| **Propionic acid** | / | / |
| **Butyric acid** | / | / |
| **Valeric acid** | / | / |
| **Malic acid** | 0.9403 g/L | 0.7209 g/L |
| **Lactic acid** | / | 8.6263 g/L |
| **Acetic acid** | / | 6.5125 g/L |
| **Citric acid** | 0.7487 g/L | 3.2443 g/L |
| **Succinic acid** | / | 0.4172 g/L |
